# PATTERNS OF UNMET NEEDS AND STRENGTHS AMONG STROKE PATIENTS AND THEIR INFLUENCE ON OUTCOMES

**DOI:** 10.1093/geroni/igz038.2704

**Published:** 2019-11-08

**Authors:** Amanda Woodward, Anne K Hughes, Mat Reeves, Michele Fritz, Paul Freddolino, Constantinos Coursaris, Sarah J Swierenga

**Affiliations:** Michigan State University, East Lansing, Michigan, United States

## Abstract

The transition home after a stroke is a particularly challenging time for patients and caregivers. There is extensive literature on unmet needs among stroke patients, but there are fewer studies that examine the strengths and resources of stroke patients and how these may influence their subsequent recovery. This study uses data from the Michigan Stroke Transitions Trial (MISTT), a pragmatic 3-arm clinical trial that tested the efficacy of case management against usual care. Two intervention groups (n=160) received up to 90 days of services from a social work case manager. A complete biopsychosocial assessment was conducted approximately 5 days after discharge. Latent class analysis was used to identify different classes based on six indicators of unmet needs and twelve indicators of strengths. Four homogenous classes were identified in the final model. Class 1 (n=56, 35%) included patients with few needs and strong social support, but few other strengths. Class 2 (n=46, 29%) had few needs and a high level of strengths across all indicators. Class 3 (n=39, 24%) has moderate needs related to mental health, non-stroke physical health, and finances, but few strengths. Class 4 (n=19, 12%) has moderate needs related to social support, non-stroke related physical health, and finances, but with moderate strengths related to indicators like coping, cognition, and insight. Class membership was related to short-term quality of life. Understanding the combination of needs and strengths has potential implications for services provided during care transitions as well as the policies and funding mechanisms that support those services.

